# Panel‐based targeted exome sequencing reveals novel candidate susceptibility loci for age‐related cataracts in Chinese Cohort

**DOI:** 10.1002/mgg3.1218

**Published:** 2020-04-26

**Authors:** Jian-Kang Li, Li‐Li Li, Wei Li, Zi‐Wei Wang, Feng‐Juan Gao, Fang-Yuan Hu, Sheng‐Hai Zhang, Shou-Fang Qu, Jie Huang, Lu-Sheng Wang, Ji-Hong Wu, Fang Chen

**Affiliations:** ^1^ Dept of Computer Science City University of Hong Kong Kowloon Hong Kong; ^2^ BGI‐Shenzhen Shenzhen China; ^3^ Guangdong Provincial Key Laboratory of Human Disease Genomics Shenzhen Key Laboratory of Genomics BGI-Shenzhen Shanghai China; ^4^ National Institutes of food and drug Control (NIFDC) Beijing P. R. China; ^5^ BGI Education Center University of Chinese Academy of Sciences Shenzhen China; ^6^ Eye Institute, Eye and ENT Hospital College of Medicine Fudan University Shanghai China; ^7^ Shanghai Key Laboratory of Visual Impairment and Restoration, Science and Technology Commission of Shanghai Municipality Shanghai China; ^8^ Key Laboratory of Myopia Ministry of Health Shanghai China

**Keywords:** age‐related cataracts, panel‐based targeted exome sequencing, susceptibility

## Abstract

**Background:**

Age‐related cataracts (ARC) is the most common blinding eye disease worldwide, and its incidence tend to become younger. However, the relationship between genetic factors and mechanisms is not fully understood. The aim of the study was to further clarify the relationship between ARC and genetic mechanisms in East Asian populations and to elucidate the pathogenesis.

**Methods:**

The study collected 191 sporadic cataracts and 208 healthy people from the eastern provinces of China, with an average age of about 60 years. All participants were subjected to a comprehensive ophthalmic clinical examination and peripheral blood samples were collected and their genomic DNA was extracted. Mutations were screened among 792 candidate genes to enhance understanding of the disease through targeted capture and high‐throughput sequencing.

**Results:**

We identified novel candidate susceptibility gene, which may serve as a potential susceptibility factor leading to an increase in the incidence of age‐related cataracts. Three novel loci are associated with age‐related cataracts significant significance: rs129882 in *DBH* (*p* = 5.27E‐07, odds ratio = 3.9), rs1800280 in *DMD* (*p* = 2.85E‐06, odds ratio = 1.4) and rs2871776 in *ATP13A2* (*p* = 4.18E‐05, odds ratio = 0.04). Gene–gene interaction analysis revealed that the most significant interactions between genes include the interaction between *DBH* and *TUB* (rs17847537 in *TUB*, rs129882 in *DBH*, *p*‐value = 2.12E‐14), and the interaction between *DBH* and *DMD* (rs1800280 in *DMD*, rs129882 in *DBH*, *p*‐value = 2.12E‐14). Pathway analysis shows that the most significant processes are concentrated in response to light stimulation (adjusted *p*‐Value = 5.56E‐03), response to radiation (adjusted P‐Value = 5.56E‐03), abiotic stimulus (adjusted *p*‐Value = 5.56E‐03). eQTL analysis shows that *DBH* rs129882 could regulate the expression of *DBH* mRNA in various tissues including retina.

**Conclusion:**

Our study indicates rs129882 and rs1800280 loci are associated with age‐related cataracts, which enlarge the gene map of age‐related cataracts.

## Graphical abstract

We identified novel candidate susceptibility gene, which may serve as a potential susceptibility factor leading to an increase in the incidence of age‐related cataracts. Our study indicates rs129882 and rs1800280 loci are associated with age‐related cataracts, which enlarge the gene map of age‐related cataracts.

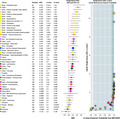

## 1 INTRODUCTION

Cataracts has become the world's first blind eye disease, accounting for about 80% of senile blindness, with age‐related cataract (ARC) being the most common type and its probability will increase as the population ages (Tang, Shentu, Tang, Ping, & Xiao‐Ning, 2019; West, 2007). Although operation is currently the most effective cataract therapy over the last few decades, challenges remain in fighting cataract across the world, including financial pressure to the health care system (America, 2007), uneven distribution of medical resources, and development of posterior capsular opacification after surgery. If the onset or progression of cataract is delayed by 10 years, the burden of cataract surgery is estimated to decrease by 50% (Hunter, Angelicheva, Levy, Pueschel, & Kalaydjieva, 2001). So, a deeper understanding of the pathogenesis and genetic etiology of age‐related cataract formation contributes to better prevention and control of the disease.

The pathogenesis of ARC is not fully understood. It is currently considered to be an multifactorial ophthalmic disease caused by both genetic and environmental variations. ARC is reportedly related to multiple environmental risk factors, from degenerative processes or personal characteristics to environmental and dietary factors, including age, gender, smoking, blood pressure, diabetes, myopia, and exposure to sunlight, among others (Tang et al., 2019).

Genetic factors also play a vital role in the formation of ARC, genetic variation may directly participate in the occurrence of ARC, and may also increase the sensitivity of the lens to environmental risk factors, leading to the occurrence of ARC (Hammond, Duncan, Snieder, Lange, & Gilbert, 2001; Su et al., 2013). Camparing to congenital cataracts, age‐related cataracts have been found associated with relatively fewer gene or loci with a clear pathogenesis, currently. Perhaps owing to their complex genetic patterns and late‐onset making it more difficult to study. GALK1 was first reported to be associated with Japanese age‐related cataract patients (Tang et al., 2019). Until now, over 40 different genes and loci have been identified associated with ARC, including *OGG1*, *EPHA2*, *GJA8*, *GALT*, *HSF4*, *CRYAA*, *GSTM1*, and *SLC16A12* (Jiang et al., 2013; Liao, Ye, Liu, & Ye, 2015; Yang et al., 2013; Zhang et al., 2016) and more. These genes show a wide range of associations. One possible similarly hypothesize that mutations that severely disrupt homeostatic functions such as in ion channels or glycemic control, might cause congenital cataract, but those that simply stress the system may cause ARC (Shiels & Hejtmancik, 2016).

To reveal the genetic loci of age‐related cataracts, we collected 191 sporadic cataracts and 208 healthy individuals from eastern China, and we performed an association study with 792 candidate genes. Our findings will further clarify the pathogenesis of age‐related cataracts and provide evidence for existing hypotheses.

## 2 METHODS

### 2.1 Sample collection

We reviewed the medical records of age‐related cataract patients diagnosed at the Eye and ENT Hospital of Fudan University from June 2016 to December 2018. According to clinical diagnostic criteria for ophthalmic examination, 191 ARC patients and 208 healthy individuals underwent clinical diagnosis of congenital cataract. All participants are required to undergo a complete eye examination. Comprehensive history and physical examination for these participating subjects were performed at length to identify both personal or family medical histories of visual impairment and other clinical abnormalities. All 208 healthy individuals showed no symptoms of cataract after detailed clinical examination, and 191 sporadic patients had symptoms of cataracts, which were diagnosed as age‐related cataracts by professional ophthalmologists. The study followed the Helsinki Declaration and was approved by the Ethics Committee of the Eye and ENT Hospital of Fudan University. Informed consent was signed by all patients or their families, with the underaged subjects's guardians signed the informed consent.

### 2.2 Targeted Gene Capture and Next‐Generation Sequencing (NGS)

Customized gene capture chip‐based next generation sequencing (NGS) was designed to encompass all the coding exons, flanking intronic regions and untranslated regions (UTRs) of 792 genes involved in common inherited eye diseases (Table S1). The Target_Eye_792_V2 chip was custom‐designed and produced by BGI (BGI‐Shenzhen,China) (Li et al., 2019). The genomic DNA sample of the proband was subject to analysis using panel‐based NGS. Whole blood from recruited members of this study was stored in an EDTA blood collection tube and genomic DNA was extracted using FlexiGene DNA Kit (Qiagen, Venlo, The Netherlands) according to the standard manufacturer's protocols. Polymerase chain reactions were done using custom primers targeting all open reading frames and the flanking intronic sequences for direct sequencing on genetic sequencer. The DNA fragment was amplified by PCR and hybridized to a DNA capture probe specifically designed for the target gene. The captured DNA fragment was eluted, amplified again, and NGS was performed using a sequencing system on MGISeq2000 Platform (MGI; Inc., Shenzhen, China) (Table S1–S4).

### 2.3 Bioinfomatic analysis

Associations between ARC and SNPs were estimated by sequence kernel association test (SKAT) (Lee, Abecasis, Boehnke, & Lin, 2014; Wu et al., 2011), which is an effective method to detect association of the sequencing data to disease phenotypes. W‐test method was used to evaluate SNP–SNP interactions (Wang et al., 2016). A SNP or an interaction pair was significant if its *p*‐value was smaller than Bonferroni‐corrected alpha of 5%. We used Cytoscape (Shannon, 2003) to draw Gene–Gene interaction network by the result of W‐test. And we used m‐code (Bader & Hogue, 2003), which was a tool of Cytoscape, to implement sub‐network cluster analysis. Expression quantitative trait loci (eQTL) analysis was carried out using the Genotype‐Tissue Expression database (Lonsdale et al., 2013).

## 3 RESULT

### 3.1 Patient characteristics, quality control, and SNP screening

We collected 399 samples, including 191 age‐related cataracts patients and 208 controls. The average age of the case group and the control group were 64 years and 63.4 years, respectively, and the men in the case group and the control group were 41.9% and 51.5%, respectively. After the quality control step, we collected a total of 12,633 SNPs from 789 genes, and we screened the data for the East Asian population with a small allele frequency (MAF) of less than 0.01, and then we obtained 2067 snps. We performed a Hardy–Weinberg equilibrium test on these data, and all SNPs showed a *p*‐value > 0.05 after Bonferroni correction.

### 3.2 Novel associations with age‐related cataracts

**Figure 1:**
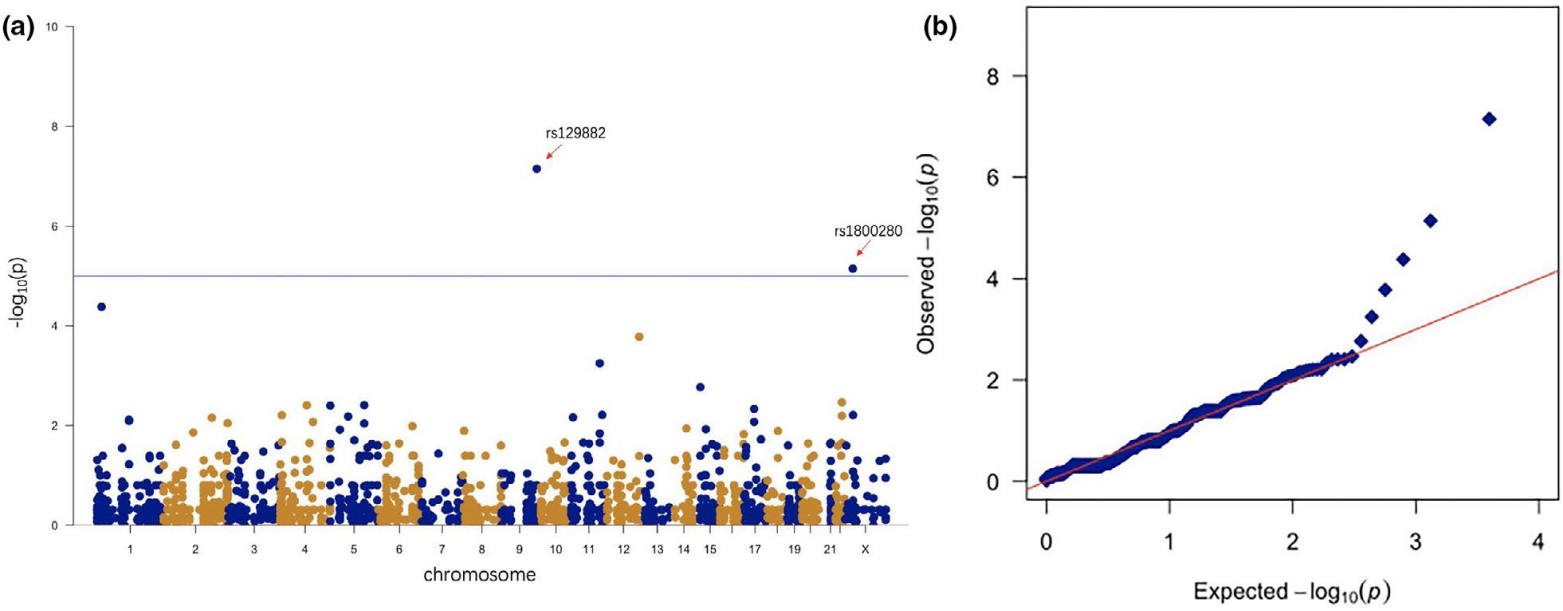
(a) Manhattan plot for the association study of 2067 locus. *p*‐values in–log10 scale are plotted against their chromosomal locations. The blue horizontal line indicates the significant level of *p* value after Bonferroni correction (*p* = 2.5 × 10^–5^). The arrow indicates *DBH* rs1298882 and *DMD* rs1800280, which had a high risk with age‐related cataract (*p* = 5.27 × 10^–7^ and 2.85 × 10^–6^). (b) QQplot for association result. It can be seen that most loci are in accordance with normal distribution, only a few of loci with strong significant is deviating from the straight line of normal distribution

Genome‐wide association analysis was performed by the SKAT method (Hasegawa, Fujimori, Takahashi, Yokohata & Masui, 2016). Using Bonferroni correction method, we detected two loci with significant levels (Armstrong, 2014; Ranstam, 2016). The first locus is rs129882 located on the *DBH* gene (C > T, *p* = 5.27 × 10–7, odds ratio = 3.9), and the rs129882 have higher OR ratios, indicating that this mutation had a higher disease risk. The second locus is rs129882 located on the *DMD* gene (C > T, *p* = 2.85 × 10–6, odds ratio = 1.4). The rs129882 located on the *ATP13A2* gene (C > T, *p* = 4.18 × 10–5, odds ratio = 0.04) also shows a strong association, whose *p*‐value was closed to the significance level. The regional analysis shows that there were no loci had a strong linkage with these loci inside the gene (Figure S1). All these three loci meet the Harvard–Weinberg equilibrium, and the P value is significant after the Bonferroni correction. The Manhattan plot of all loci is shown in Figure 1a and QQplot is shown in Figure 1b. Table 1 shows the top ten significant loci.

**Table 1:** Top ten significant susceptibility loci in association analysis. The threshold for the *p* value after bonferroni correction was 2.5 × 10^–5^

Rank	SNP	Chr	Pos	Description	Gene	*p*‐value	OR（95% CI）	MAF
1	rs129882	chr9	133,658,547	C > T	*DBH*	5.27E‐07	3.9 (2.4,6.3)	0.36
2	rs1800280	chrX	31,478,233	C > T	*DMD*	2.85E‐06	1.4 (1.0,1.9)	0.07
3	rs2871776	chr1	17,001,806	T > C	*ATP13A2*	4.18E‐05	0.04 (0,0.7)	0.29
4	rs1154510	chr12	121,857,429	T > C	*HPD*	1.55E‐04	1.5 (1.0,2.4)	0.14
5	rs609261	chr11	108,287,407	T > C	*ATM*	5.68E‐04	0.06 (0,1.3)	0.39
6	rs1800414	chr15	27,951,891	T > C	*OCA2*	1.87E‐03	2.8 (1.6,4.8)	0.4
7	rs147244947	chr22	41,117,770	C > G	*EP300*	3.42E‐03	15.5 (0.9,27.4)	0.02
8	rs154001	chr5	128,349,443	C > T	*FBN2*	3.74E‐03	1.4 (1.1,2.0)	0.06
9	rs1800804	chr4	99,574,660	T > C	*MTTP*	3.94E‐03	15.6 (0.9,27.1)	0.14
10	rs10069690	chr5	1,279,675	C > T	*TERT*	4.14E‐03	1.3 (0,82.5)	0.15

### 3.3 Gene–Gene interaction of ARC related susceptibility genes

**Figure 2:**
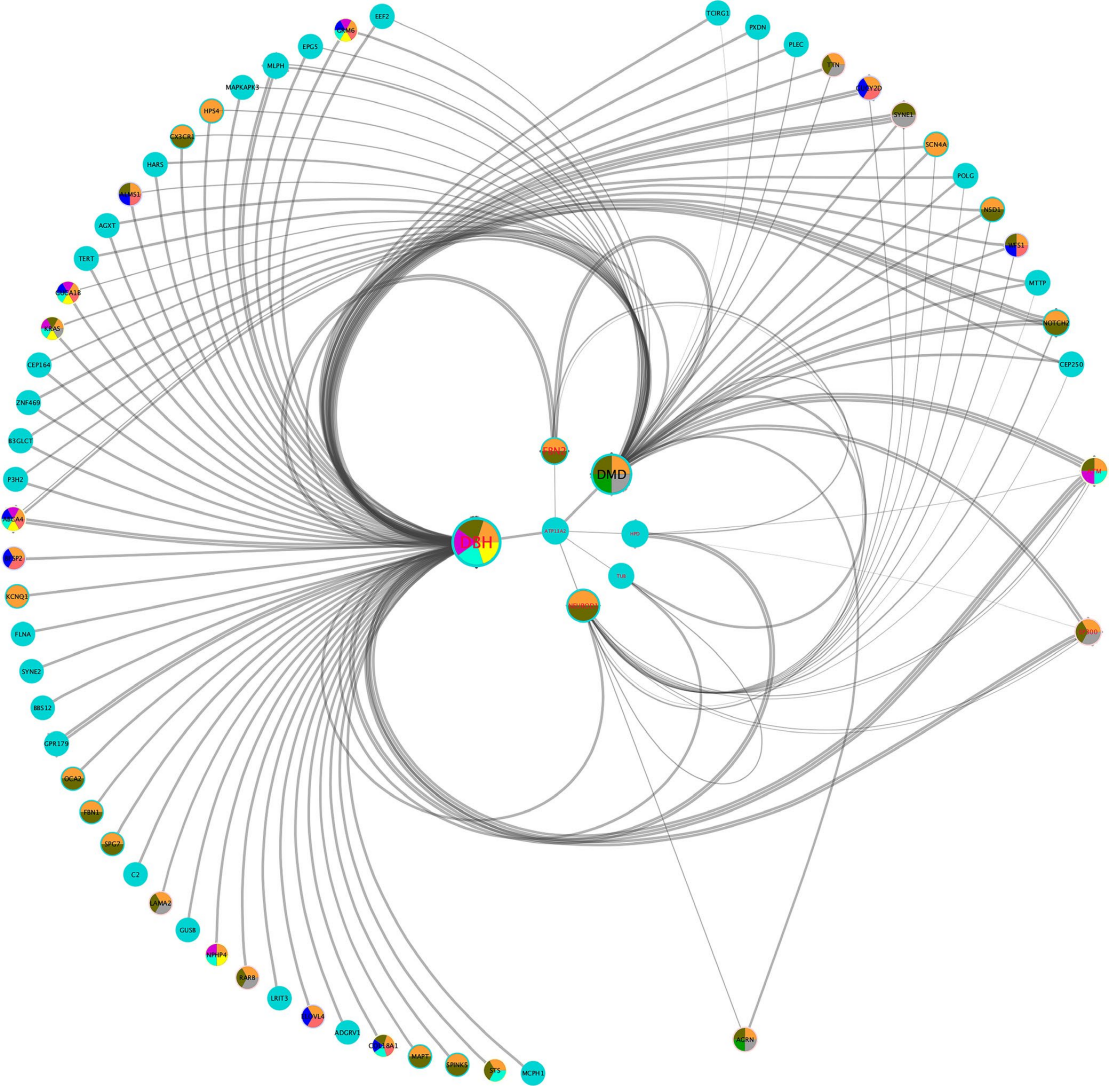
Gene–gene interaction network visualizes the results of the w‐test. Bonferroni revises the significance threshold to 1.3 × 10^–6^, and different colors represent different GO terms where genes in networks are enriched. The fonts highlighted in red represent the significant sub‐networks analyzed by M‐code

W‐test method was used to conduct pairwise detection to find the interaction between the loci. We calculate the epistasis between the SNPs, and their significance in SKAT analysis was less than the threshold. From all 2067 SNPs, we set a threshold of 0.1 and 195 SNPs were included. Among them, a total of 38,025 SNP pairs were formed and 62 pairs of loci past the significance threshold of *p* < .05 after bonferroni correction. *DBH* gene have most of the interactions (62 gene pairs), while *DMD* and neuroD1 genes also have extensive interactions (46 gene pairs and 20 gene pairs, respectively). The most significant interactions between genes include the interaction between *DBH* and *TUB* (rs17847537 in *TUB*, rs129882 in *DBH*, *p*‐value = 2.12E‐14), and the interaction between *DBH* and *DMD* (rs1800280 in *DMD*, rs129882 in *DBH*, *p*‐value = 2.12E‐14). The 20 SNP pairs with the most significant *p*‐value are listed in Table 2. A network of gene–gene epistasis interaction was formed by Cystoscape using the significance result of W‐test (Figure 2).

**Table 2:** The top 20 SNP pairs are detected by W‐test in 191 patients

rank	rsID1	position1	gene1	MAF1	rsID2	position2	gene2	MAF2	*p*‐value
1	rs17847537	C > T	*TUB*	0.3611	rs129882	C > T	*DBH*	0.0258	4.66E‐15
2	rs129882	C > T	*DBH*	0.7034	rs1800280	C > T	*DMD*	0.3611	2.12E‐14
3	rs55677134	C > T	*TTN*	0.3611	rs129882	C > T	*DBH*	0.0288	2.52E‐14
4	rs2871776	T > C	*ATP13A2*	0.3611	rs129882	C > T	*DBH*	0.7321	2.59E‐14
5	rs154001	C > T	*FBN2*	0.3611	rs129882	C > T	*DBH*	0.9167	3.17E‐14
6	rs75523528	T > A	*P3H2*	0.3611	rs129882	C > T	*DBH*	0.0159	3.92E‐14
7	rs1801208	G > A	*WFS1*	0.3611	rs129882	C > T	*DBH*	0.0794	4.00E‐14
8	rs117579809	G > T	*HARS*	0.3611	rs129882	C > T	*DBH*	0.0188	9.89E‐14
9	rs35126034	A > T	*HPS4*	0.3611	rs129882	C > T	*DBH*	0.0169	1.16E‐13
10	rs3732379	C > T	*CX3CR1*	0.3611	rs129882	C > T	*DBH*	0.0278	1.78E‐13
11	rs76894284	G > A	*SCN4A*	0.3611	rs129882	C > T	*DBH*	0.0188	1.85E‐13
12	rs3092856	C > T	*ATM*	0.3611	rs129882	C > T	*DBH*	0.0149	2.07E‐13
13	rs191142743	C > T	*GRM6*	0.3611	rs129882	C > T	*DBH*	0.0139	2.62E‐13
14	rs147244947	C > G	*EP300*	0.3611	rs129882	C > T	*DBH*	0.0123	2.71E‐13
15	rs2305111	G > T	*EEF2*	0.3611	rs129882	C > T	*DBH*	0.0149	3.00E‐13
16	rs140494095	G > A	*EPG5*	0.3611	rs129882	C > T	*DBH*	0.0159	3.55E‐13
17	rs609261	T > C	*ATM*	0.3611	rs129882	C > T	*DBH*	0.3948	3.88E‐13
18	rs80292002	A > G	*MLPH*	0.3611	rs129882	C > T	*DBH*	0.0298	3.89E‐13
19	rs1800804	T > C	*MTTP*	0.3611	rs129882	C > T	*DBH*	0.1359	5.56E‐13
20	rs150941761	A > C	*EP300*	0.3611	rs129882	C > T	*DBH*	0.0123	6.01E‐13

Next, GO terms analysis is conducted to further illustrate the interaction between genes (Teng et al., 2013). The 10 most prominent Go terms are shown in table with different colors. The results of gene enrichment analysis shows that the most significant processes are concentrated in eye‐related biological processes, such as sensory perception of light stimulus (suid = 15,810, adjusted *p*‐Value = 1.20E‐04) and visual perception (suid = 15,810, adjusted *p*‐Value = 1.20E‐04).

*DBH* gene is significantly enriched in response to light stimulation (SUID = 15,809, adjusted *p*‐Value = 5.56E‐03), radiation (SUID = 15,601, adjusted *p*‐Value = 5.56E‐03), abiotic stimulus (SUID = 15,639, adjusted *p*‐Value = 5.56E‐03) and other biological processes, which is consistent with the previously reported age‐related cataract susceptibility factors in the population in eastern China. *DMD* gene is significantly enriched in muscle structure development (SUID = 15,552, adjusted *p*‐Value = 4.26E‐03), neurotransmitter receptor metabolic process (SUID = 15,557, adjusted *p*‐Value = 4.52E‐03), and anatomical structure development (SUID = 48,856, adjusted *p*‐Value = 5.69E‐03) which may indicate that the mutation of *DMD* gene affects the relevant development process and thus affects the incidence of age‐related cataract patients.*ATP13A2* gene does not appear to be significantly enriched in any biological process, its mutations may be widely involved M‐code was used to analyze the sub‐network in gene–gene epistasis interaction network. *DBH*, *ATP13A2*, *NEUROD1*, *FBN2* and *HPD*, *EP300*, *ATM* has been found out to form a sub‐network, which indicates that these genes have a stronger and complex interaction relationship with each other. Go enrich analysis shows these gene collaboratively involved in homeostatic process, regulation of biological quality pathways in various biological processes by regulating the expression of ATPase.

### 3.4 rs129882 regulated the expression of *DBH*

**Figure 3:**
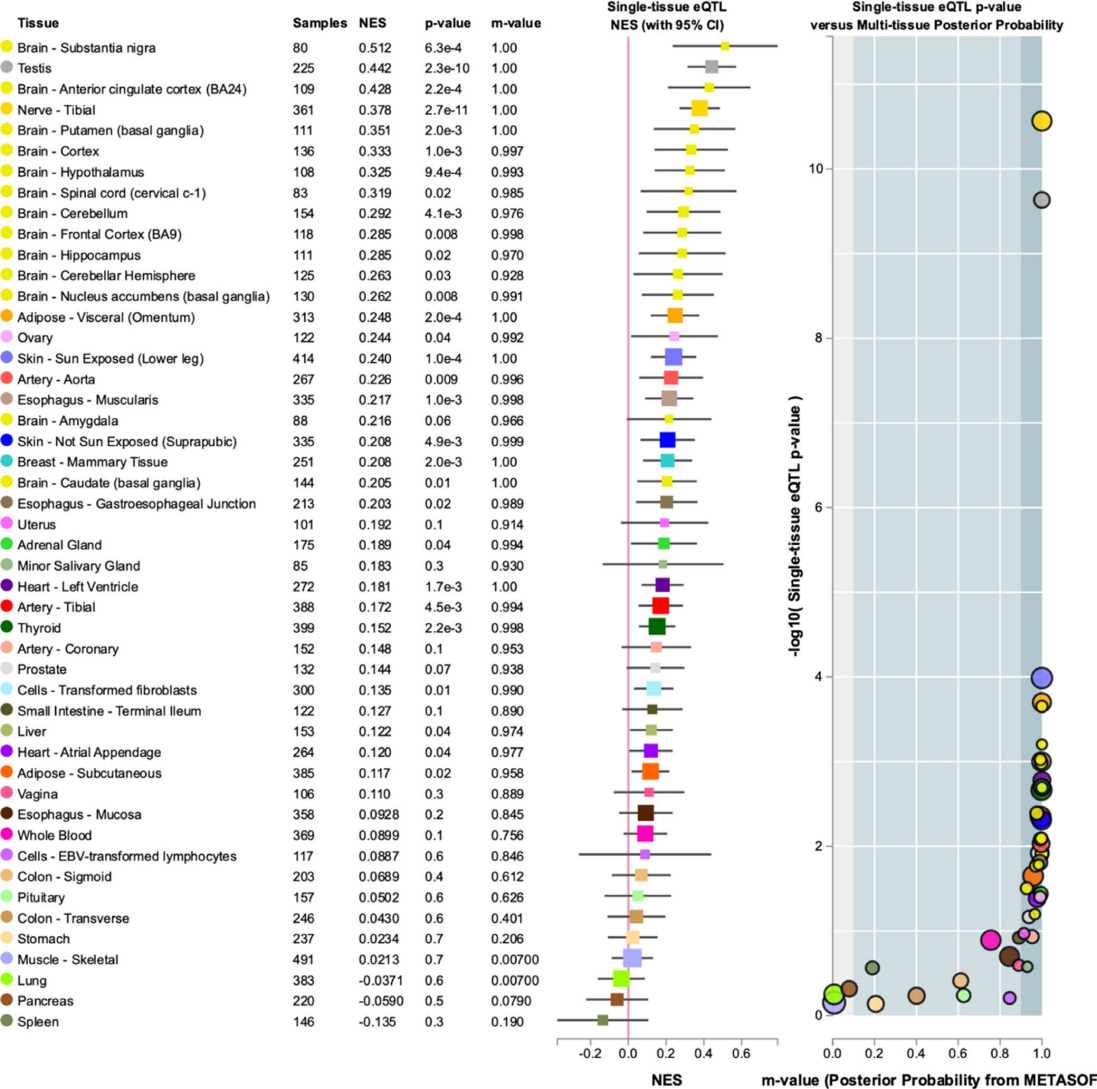
eQTL analysis shows that *DBH* rs129882 could regulate the expression of *DBH* mRNA in various tissues

We checked significant variant of association study with mRNA expression, rs1800280 in *DMD*, rs2871776 in *ATP13A2* show no correlation with gene expression in eQTL analysis (Zeng et al., 2019). rs129882 in *DBH* shows significantly associated with multiple organizations in eQTL analysis, significant genes differential expression tissues including brain tissue (*p* = 2.7 × 10–11, NES = 0.38), testis tissue(*p* = 2.3 × 10–10, NES = 0.44), Brain‐Cerebellum tissue (*p* = 8.3 × 10–8, NES = −0.34), Brain‐Caudate tissue(*p* = 1.8 × 10–6, NES=−0.32), Brain‐Cortex(*p* = 2.2 × 10–6, NES = −0.36), and Brain‐Frontal Cortex tissue(*p* = 8.1 × 10–6, NES = −0.31). And it can be seen that T > C in rs129882 will up regulate the expression of *DBH* in these tissues. We also checked data in GTEx's ophthalmic data set (Ratnapriya et al., 2019), rs129882 shows significant association between variant and DBH's expression in retina (*p* = .0144297) (Figure 3).

## 4 DISCUSSION

*DBH* gene is related to dopamine synthesis (Punia et al., 2010). *DBH* protein expression was substantially decreased with age in the retina, the retina may be more sensitive to age‐related loss of sympathetic neurotransmission than the choroid, which may partially explain normal age‐related vision loss in the elderly (Smith, Sharma, & Steinle, 2007). Previous reports show that rs129882 mutation is significantly associated with Parkinson's disease and attention hyperactivity (Algamal, Alhamzawi, & Ali, 2018; Punia et al., 2010; Tong et al., 2015). To date, no studies have shown that rs129882 was associated with age‐related cataracts.

The rs129882 locus is located in the UTR3 region of *DBH* gene, and has no effect on the structure of the protein. However, GTEx data show that the mutation can affect the expression of *DBH* gene in multiple tissues, including the retina. And the result of regional analysis shows that rs129882 locus does not link with other *DBH* gene loci in our data, which may indicate that rs129882 may affect the expression of *DBH* gene by its own effect rather than linked to other loci. Recent studies have shown that the 3'UTR of *DBH* could play a vital role in the recruitment of transcription factors to activate transcription of *DBH*‐AS1 and therefore interfere with expression, and the substitution of the C to T allele of rs129882 activates the transcription of *DBH*‐AS1 which reduces/represses the expression level of *DBH* (Smith et al., 2007).

Environment is also a risk factor for rs129882 mutation of *DBH*. Our analysis showed that the rs129882 and its interacting loci are concentrated in the GO term related to light reactions. Previous study on age‐related cataracts in eastern China showed that age‐related cataracts are related to environmental factors such as radiation and light stimulation, which is consistent with our findings (Wang et al., 2012). All these finding suggest that rs129882 may affect the expression of *DBH* gene, which in turn affects human stimulation of radiation and light response, thereby increasing the risk of developing age‐related cataracts.

*DMD* is the largest known human gene, which encode a protein called dystrophin located primarily in muscles used for movement (skeletal muscles) and in heart (cardiac) muscle. Gene function and regulation of the *DMD* gene outside of muscular tissue is far more perplexed. *DMD* expression in the human retina is required for normal function and its products have been reported related to regulate retinal function and vascular morphology in response to age and retinal ischemia (Bucher et al., 2019; Pillers et al., 1993). A common complication of Duchenne muscular dystrophy after glucocorticoid treatment is cataracts (Rice, Wong, Horn, & Yang, 2018). rs1800280 in *DMD* locus is another cataract‐related loci we found. The C > T alter is a missense mutation and change in the 2,937 amino acid from Gln to Arg. rs1800280 was seen as a benign mutation in *DMD* and *BMD* disease. The slight mutation of *DMD* gene possibly leads to risk accumulating of retina damage with aging. To the best of our knowledge, this study presents the first potential evidence of an association between *DMD* mutation and age‐related cataract.

*ATP13A2* belongs to the P‐type superfamily of ATPases that transport inorganic cations and other substrates across cell membranes. It has been reported that mutations in *ATP13A2* cause abnormal accumulation of cations, leading to disease. rs2871776 is an intron mutation in *ATP13A2* and has not been linked to eye diseases. Gene–gene interaction analysis showed that *ATP13A2* has complex and powerful interactions with other genes and has a strong GWAS significance, but *ATP13A2* has not enriched to same pathway with other genes. The pathogenesis of *ATP13A2* and ARC needs to be further explored. We speculate that other genetic and risk factors that alter the physical and chemical environment of the retina will enhance the accumulation of cations caused by *ATP13A2* mutations.

The pathogenesis of ARC is not fully understood. We found that most mutations are related to homeostatic processes, not directly to lens proteins or lens cells. Unlike hereditary cataracts, even minor mutations (with cumulative effects of different mutations and mutation products that accumulate with age) can cause disease, suggesting that ARC is very heterogeneous.

The results of our research may support the hypothesis. Most of the mutation we found were associated with homeostatic process rather than crystallin or lens cell directly. Unlikely to the hereditary cataracts, even slight mutations, with the cumulative effect of different mutations and the accumulation of mutation product with aging, is possible to cause disease, which indicates that ARC is extremely heterogeneous. Above all, a large population study is needed to clarify the complex genetic and environmental influence on ARC and contribute to our understanding of pathogenesis to these disease.

## CONFLICT OF INTEREST

The authors declare that the research was conducted in the absence of any commercial or financial relationships that could be construed as a potential conflict of interest.

## Supporting information

Supplementary Material

## Data Availability

The data that support the findings of this study have been deposited in the CNSA (https://db.cngb.org/cnsa/) of CNGBdb with accession code CNP CNP0000503
