# Supplementary material for: Panel‐based targeted exome sequencing reveals novel candidate susceptibility loci for age‐related cataracts in Chinese Cohort
Source: Mol Genet Genomic Med. 2020 Apr 26;8(7):e1218. doi: 10.1002/mgg3.1218 (PMC7336732; doi:10.1002/mgg3.1218)

Table S1. Demographic data of study participants.

Table S2. Depth and coverage of targeted capture sequencing.

Table S3. Quality control information.

Table S4. Gene list of capture panel.

Figure S1. Regional analysis of these three genes.


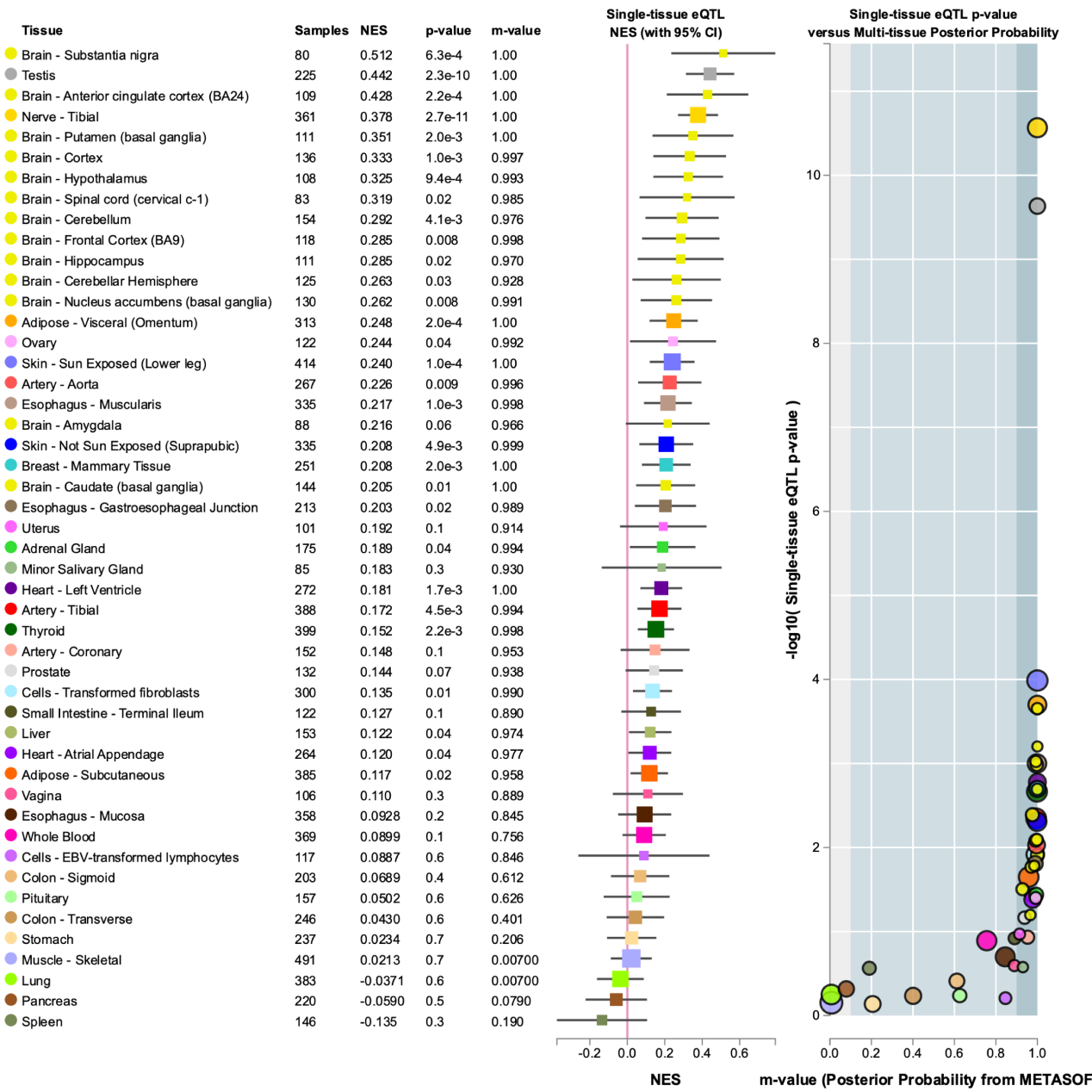

Supplement: Supplementary file 1 — Supplementary Material [file MGG3-8-e1218-s001.docx]
