# Supplementary material for: Panel‐based targeted exome sequencing reveals novel candidate susceptibility loci for age‐related cataracts in Chinese Cohort
Source: Mol Genet Genomic Med. 2020 Apr 26;8(7):e1218. doi: 10.1002/mgg3.1218 (PMC7336732; doi:10.1002/mgg3.1218)
Supplement: Supplementary file 2 [file MGG3-8-e1218-s002.pdf]

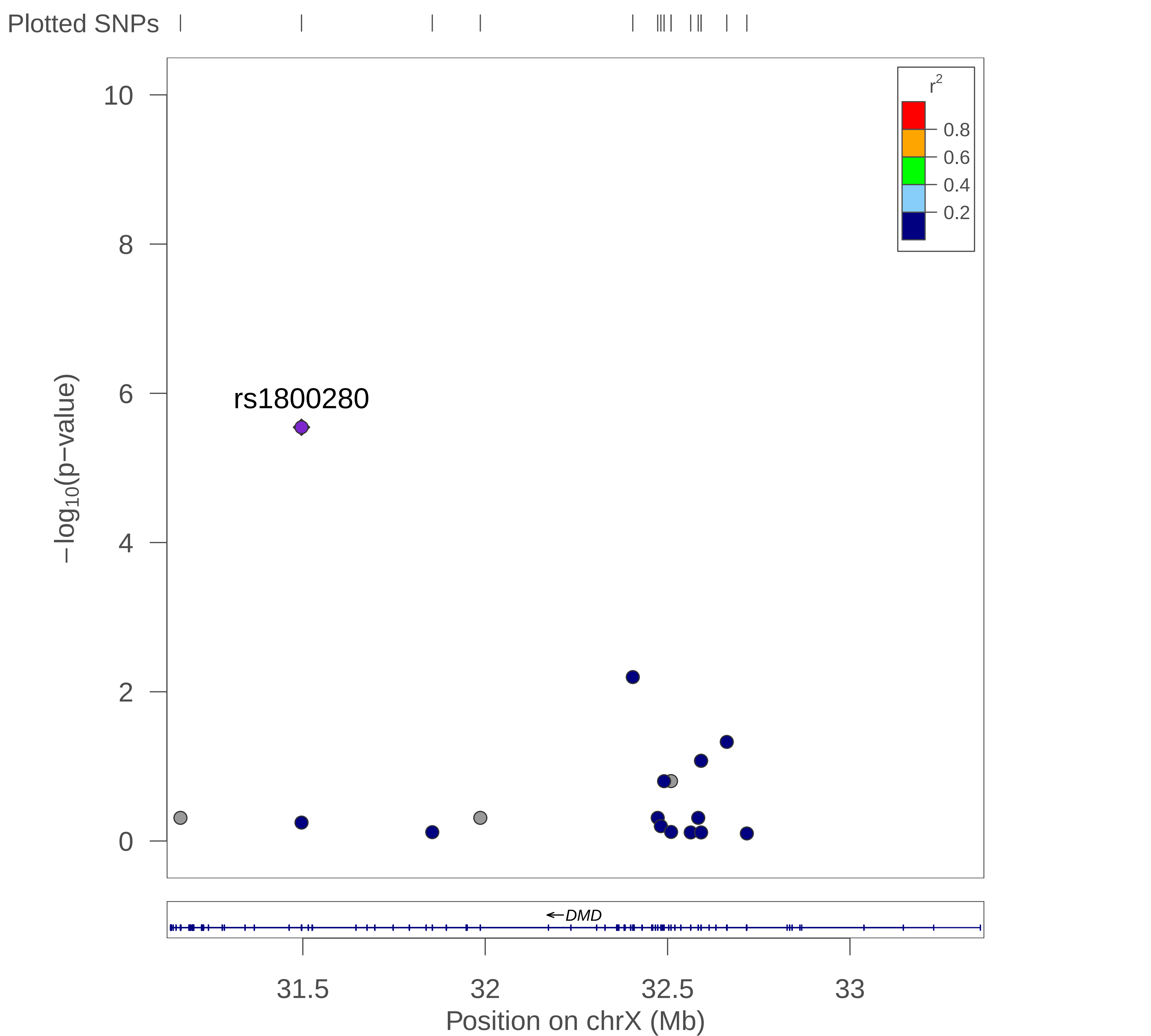
Plotted SNPs
rs1800280
r2
0.8
0.6
0.4
0.2
10
8
6
4
2
0
−log10(p−value)
DMD
31.5
32
32.5
33
Position on chrX (Mb)

date:	Sun Jun 2 03:35:28 2019
build:	hg19
display range:	chr23:31127344−33367726 [31127344−33367726]
hilite range:	0 − 0 [0 − 0]
reference SNP:	chr23:31496350
number of SNPs plotted:	19
min sk:	2.85E−6 [chr23:31496350]
max sk:	7.92E−1 [chr23:32717258]

Make more plots at http://csg.sph.umich.edu/locuszoom/

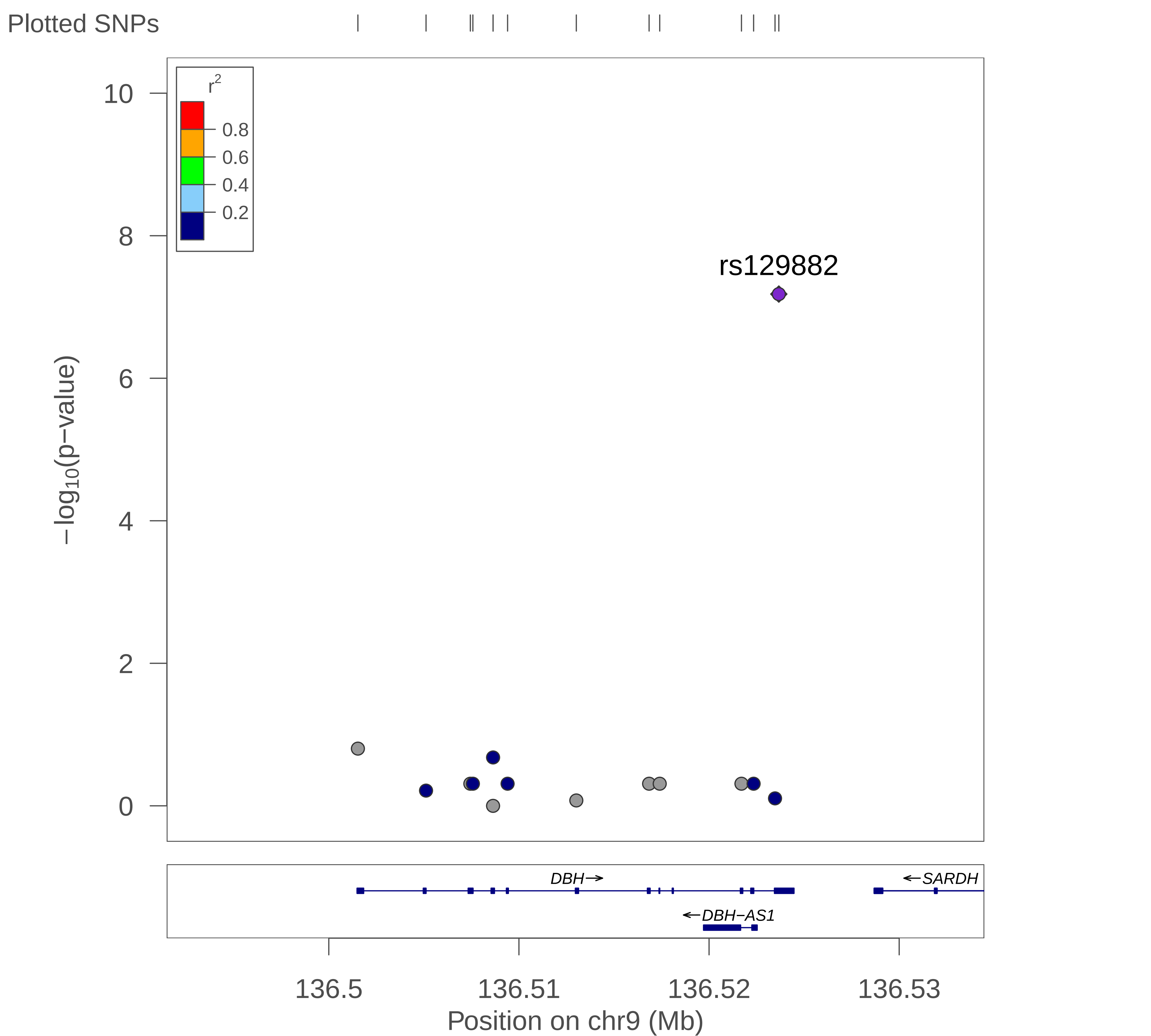

Plotted SNPs
r2
0.8
0.6
0.4
0.2
10
8
6
4
2
0
−log10(p−value)
rs129882
DBH→
←SARDH
←DBH−AS1
136.5
136.51
136.52
136.53
Position on chr9 (Mb)

date:	Mon Jun 3 00:47:04 2019
build:	hg19
display range:	chr9:136491484−136534466 [136491484−136534466]
hilite range:	0 − 0 [0 − 0]
reference SNP:	chr9:136523669
number of SNPs plotted:	15
min sk:	6.59E−8 [chr9:136523669]
max sk:	1E0 [chr9:136508639]

Make more plots at http://csg.sph.umich.edu/locuszoom/

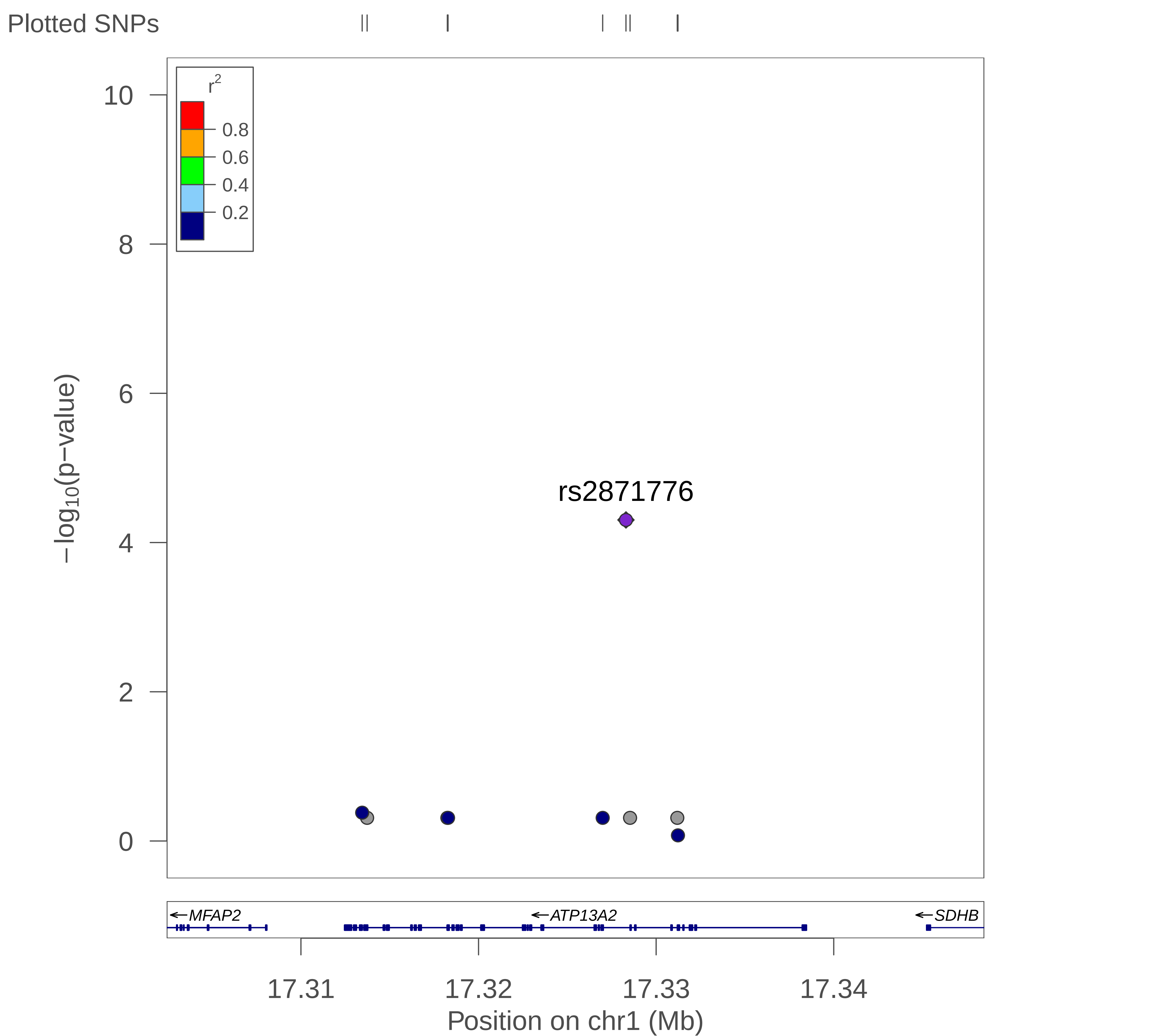

Plotted SNPs
r2
0.8
0.6
0.4
0.2
10
8
6
4
2
0
−log10(p−value)
rs2871776
MFAP2
ATP13A2
SDHB
17.31
17.32
17.33
17.34
Position on chr1 (Mb)

date:	Mon Jun 3 00:52:34 2019
build:	hg19
display range:	chr1:17302452−17348467 [17302452−17348467]
hilite range:	0 − 0 [0 − 0]
reference SNP:	chr1:17328301
number of SNPs plotted:	10
min sk:	4.98E−5 [chr1:17328301]
max sk:	8.4E−1 [chr1:17331229]

Make more plots at http://csg.sph.umich.edu/locuszoom/
